# Software Fault Localization through Aggregation-Based Neural Ranking for Static and Dynamic Features Selection

**DOI:** 10.3390/s21217401

**Published:** 2021-11-07

**Authors:** Abdulaziz Alhumam

**Affiliations:** Department of Computer Science, College of Computer Sciences and Information Technology, King Faisal University, Al-Ahsa 31982, Saudi Arabia; aahumam@kfu.edu.sa

**Keywords:** fault localization, neural ranking, parameter selection

## Abstract

The automatic localization of software faults plays a critical role in assisting software professionals in fixing problems quickly. Despite various existing models for fault tolerance based on static features, localization is still challenging. By considering the dynamic features, the capabilities of the fault recognition models will be significantly enhanced. The current study proposes a model that effectively ranks static and dynamic parameters through Aggregation-Based Neural Ranking (ABNR). The proposed model includes rank lists produced by self-attention layers using rank aggregation mechanisms to merge them into one aggregated rank list. The rank list would yield the suspicious code statements in descending order of the rank. The performance of ABNR is evaluated against the open-source dataset for fault prediction. ABNR model has exhibited noticeable performance in fault localization. The proposed model is evaluated with other existing models like Ochiai, Fault localization technique based on complex network theory, Tarantula, Jaccard, and software-network centrality measure concerning metrics like assertions evaluated, Wilcoxon signed-rank test, and Top-N.

## 1. Introduction

It is hard for developers to find and correct software flaws while the program is being developed and after its release. The complete process of bug identification is time-consuming, and the automated software does the job perfectly through fault localization. Using fault localization models reduces the debugging expenses and allows the developers to devote more to possible vulnerable components of the software. Traditionally, fault localization, which focuses on locating defects in software manually, has been a difficult, time-consuming, and costly job because of the complexity of large-scale software systems [1]. The developer’s knowledge, expertise, and judgment are required to locate and classify the snippet which is most likely to contain bugs. As a result of the constraints mentioned above, interest has been revived in creating automated models for localization of faults in software while at the same time minimizing human involvement.

The automated models are classified as static and dynamic models for fault localization [2]. The static model does consider various static features of the software, such as the log reports of the bugs, bug history reports, code modification logs, and performs the fault matching with the existing issues [3,4]. Moreover, the static models mainly recognize the errors in the code like using the dangling pointers, syntax errors, security-related access privileged errors in snippets, and the code-tempting issues [5]. The dynamic models relay the dynamic characteristics for the bug identification by introspecting the code’s response in the run-time environment. The use of test cases would assess the vulnerability severity and rank for each such code snippet in the software. However, in either of the models, the bug localization in developing robust software is quite far from a satisfactory outcome.

Fault localization is a time-consuming and laborious task for dealing with massive software projects for two primary reasons. The formal reason is that there are typically many pending faults to be still found and fixed. The well-known Eclipse bug repository has reported around 200 issues each day approaching release dates, whereas the Debian project has reported around 150 issues in a single day [6]. Another pivotal reason is the time taken for fault isolation. Gaeul Jeong [7] has observed that almost all problems take 100 to 200 days to fix in the PostgreSQL project. To repair just half of the issues takes nearly 300 days. Most issues in the Tomcat project take 40–200 days to fix. It is assumed that 5% of all problems may take almost two years to fix in the worst-case scenario.

The features are exceptionally important in the fault localization process, either static or dynamic features. When any of those features are considered alone, few challenges will make the process of fault identification challenging. When the static features are considered alone, the assessment model may fail to recognize the dynamic software features. Furthermore, the significant features and essential functional characteristics will be missing when dynamic features alone are considered. The deep neural network models efficiently consider both features with an exceptional learning capability [8]. Various studies on fault localization incorporate something into the software to track the model’s functionality and outcome [9,10]. The different fault localization techniques include programming constraints, program log reports, breakpoints, profiling. Moreover, all the fault localization tools are designed by incorporating all these features for the effective debugging of the software.

Program constraints are assertions introduced to a program that must be true to function correctly throughout its execution. These constraints are generally specified in the program as a conditional statement, which causes the program to stop execution if any constraint is not satisfied [11]. Program log [12] inserts are often included with the code on a hit-or-miss basis to check variables and other program information updating processes that utilize stored log files or printed run-time information to identify failure when they see strange program behavior. Using the breakpoints [13] is the other approach used in fault localization. It will halt the program whenever it approaches a pre-set spot, enabling the programmer to check current conditions. The programmer may alter the code and resume the program’s execution to monitor the response of the code with a possible bug. The run-time profiling [14] of the program performance indicators such as execution speed and memory consumption is known as profiling. It is often used to validate the code for the issues like memory leaks, unexpected execution patterns, and program performance evaluations.

The contributions through the current studies are accomplished over multiple phases, including identification of the features used for recognizing the possible vulnerable statements in the software program. The statements are provided with an initial vulnerability rank concerning the global rank of the statements in the program. The ranks are automatically assigned to each statement in the program by tokenizing the statement. The ranks are further optimized through an aggregation-based neural ranking model that will assist the programmer’s debugging and fault localization much easily and conveniently.

The entire paper is organized on the following grounds. Section 1 presents the introduction to the study on fault localization, Section 2 presents the brief literature about the existing models, and Section 3 presents the background of the proposed model, which covers the feature selection model. Section 4 offers the proposed model, and Section 5 presents the proposed model’s statistical analysis concerning the other existing models. Section 6 provides the conclusion and the future perspective of the current study on bug localization.

## 2. Literature Review

There are various fault localization approaches available that are widely used in real-time fault diagnosis models. Statistic debugging models are one of the most predominantly used fault localization techniques. The bugs are identified through fault prediction snippets incorporated in the software [15] as presented in Liblit05. The model here works with the probability ρ in determining the fault in the software. The fault(ρ) denotes the correct fault prediction, and the context(ρ) denotes the code of ρ implying the fault. All the cases whose score is fault(ρ)−context(ρ)≤0 are ignored. The leftover criteria are evaluated by respective significance ratings, indicating the association between predicates and software faults. Higher-scoring predicates must be evaluated immediately [1].

The other conventional mode of bug tracking, namely the slice-based technique [16], is performed by normalizing the software code into multiple components named the segments. Each such segment is verified for the bug by evaluating the response of the snippet. One primary use of static slicing is to assist developers in finding flaws in their systems by reducing the searching space. Because a failure could be traced to something like a variable’s value being wrong, debugging can only focus on searching on the slice containing the vulnerability rather than the complete software. Using static slicing does have the drawback of including all operational snippets that may potentially influence the parameter’s value in the snippet. As a consequence, it may produce an incorrect prediction. Moreover, dynamic slicing can determine the snippet that impacts a specific value seen at a particular location rather than potentially influencing such a value.

Probabilistic and empirical causation theories drive spectrum-based fault localization models [17]. If the software fails, this log data may be employed to determine the vulnerable segment of the program. It shows which snippets of the software program under test have been examined during an operation. Program state-driven bug localization is the other most predominantly used technique that keeps track of the values and the outcomes of the snippets in the software, and periodically the values are examined for the fault localization. The faults are recognized by matching the states of the development version of program snippets with a reference version. It also changes certain parameter values to check which triggers incorrect software execution.

The techniques like MULTRIC [18], TraPT [19], FLUCCS [20], and PRINCE [21] have demonstrated that Learning-to-Rank methods can assist in the identification of fault statements by using a variety of fault-diagnostic characteristics of varying dimensions. Limited in its ability to automatically choose strong preexisting features and find new advanced features for fault localization, it may not fully use the training data information gathered. The observation over the model DeepFL, which ranks suspicious logics in the program using Multi-Layer Perceptron [22]. As a result, researchers have lately begun highlighting the best of several conventional fault localization methods using machine learning to achieve more efficiency. Moreover, sophisticated deep learning techniques are used to explore powerful features for fault localization and accurately rank suspicious program snippets. 

## 3. Background

The instances in the project’s implementation phase where unexpected behavior happens are failures, errors, and faults. A fault in the computer program is defined as any inappropriate move, procedure, or data specification, sometimes termed the bug. The developer may unintentionally land the bug into the program while writing. An error is a discrepancy between a calculated value and the actual value in a certain context. Failure is defined as a system failing to execute its task following the expectations of the developer. So, when the software fails to operate as expected, there must be a possible bug in the code, and if such bugs or faults are ignored, the complete software may fail.

The proposed model relies on extracting the features associated with software evaluation and assists in analyzing the working procedure of the software snippet. Thereby the features are ranked based on the probabilistic measure for the failure of the snippet. Later, the ranks are used to analyze the software’s design principle and work procedure closely. Both static and dynamic features are considered for performing the fault localization and the other comprehensive feature set. The use of various classes, packages, and objects are common throughout the programming languages. Objects are created at run-time, whereas other elements are static. Their association among them is mostly dynamic [23]. A few such relations are presented in Table 1.

**Figure 1:**
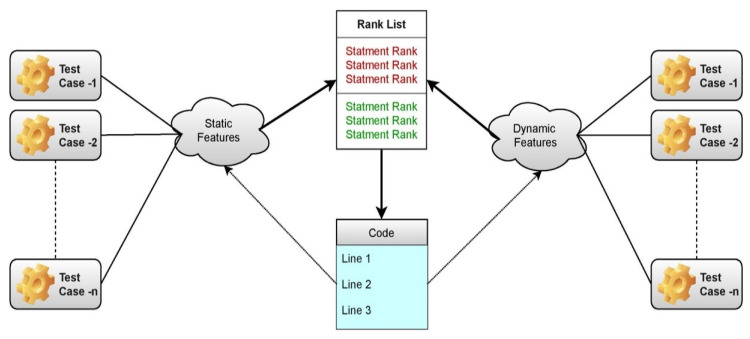
Represents the static and dynamic features in statement rank generation.

Static methods create implementation-based requirements, whereas dynamic ones generate their specifications by watching the program run. Execution-trace analyses include dynamic strategies. The ranking model’s construct is presented in Figure 1, where static and dynamic features are considered.

### 3.1. Static Feature Extraction

The static feature set involves the set of parameters in the software program that have a potential vulnerability that may lead to the bug in the code snippet or software failure. The feature set is all about the metadata about the program, which elucidates the complete structure of the program like the integer usage, conditional statements, the indentation of the expressions and various labels, and the annotations that are used across the code. Table 2 presents the complete set of static features considered in the bug localization process.

### 3.2. Dynamic Feature Extraction

The dynamic features of bug identification include the execution procedures and the outcomes associated with them. There are various bug localization mechanisms like Spectrum-Based Fault Localization [24], Predicate-based Fault Localization [25], and Program snippet analysis. The dynamic features are recognized by executing the test cases over the program, analyzing the program response to the test cases, and analyzing the stack data to trace the program’s behavior. The dynamic features and the vulnerable code snippets and statements are identified through either of the models, and all such statements are ranked through an Aggregation-Based Neural ranking mechanism.

#### 3.2.1. Spectrum-Based Fault Localization

In Spectrum-Based Fault Localization model, the possible fault and potential vulnerability ranking are assigned to each trace of program components like expressions, code statements, conditional branching statements, declarations, and assignment statements collected for each test case using a mathematical formula specific to the method. The suspiciousness rank indicates the likelihood that the statement or a snippet in the software program is defective. Using a spectrum-based fault localization strategy, every snippet’s dependency information is analyzed while running test cases. The correlation and dependency information are combined with a predetermined vulnerability analysis scheme to approximate suspicious ranks for each such program element.

The ranking is based on the number of times the test cases successfully pass and failover the number of times being executed [26]. Let us assume a program P which is considered for the assessment in the current study with a set of elements which are identified as $\left\{p_{1},p_{2},\ldots ,p_{n}\right\}$ such that $\sum_{p=1}^{n}p\in P$. The formula for rank assessment is presented in Equation (1):

(1) $$stat_{rank}\left(SBFL\right)=\frac{fail_{tc}\left(stat\right)}{\sqrt{{{tot}_{fail}}_{tc}(fail_{tc}\left(stat\right)+pass_{tc}\left(stat\right)})}$$

From Equation (1),

$stat_{rank}$—Represents the statement rank

$fail_{tc}\left(stat\right)$—Represents the fail test cases associated with the statement;

$pass_{tc}\left(stat\right)$—Represents the passed test cases associated with the statement;

$to{t_{fail}}_{tc}$—Represents the total failed test cases throughout the program.

This equation demonstrates this same fundamental concept of spectrum-based fault localization approaches: the greater the number of times failed test cases execute a statement, the greater the suspicious score of that statement; the greater the number of times a statement is executed bypassed test cases, the relatively small the suspicious score of that element; and thus illustrates the correlation among test cases and program components. The evaluated ranking model will assist in prioritizing the statement with the code snippet and assist in appropriate fault localization through the trace.

#### 3.2.2. Predicate-Based Fault Localization

The predicate-based fault localization-based fault localization approach assesses the statements ranks in the program with a set of predetermined predicates. The program is then implemented for execution, and the values of predicates gathered throughout executions are being used to rank the statements. By combing several executions, the predicate-based approach pinpoints the key predicates associated with the preliminaries of the software failure. Equation (2) determines the predicate-based ranking for the statement within the program, and the rank was determined based on the execution of the test cases.

(2) $$stat_{rank}\left(PBFL\right)=\frac{2}{\frac{1}{\alpha \left(stat\right)}+\frac{1}{\beta \left(stat\right)}}$$

where α and β are increase and sensitivity and are determined in the Equations (3) and (4):

(3) $$\alpha =\frac{fail_{tc}\left(stat\right)}{pass_{tc}\left(stat\right)+fail_{tc}\left(stat\right)}-\frac{{fail\prime }_{tc}\left(stat\right)}{{pass\prime }_{tc}\left(stat\right)+{fail\prime }_{tc}\left(stat\right)}$$

(4) $$\beta =\frac{\log\left(fail_{tc}\left(stat\right)\right)}{\log(to{t_{fail}}_{tc})}$$

From Equations (3) and (4),

${fail\prime }_{tc}\left(stat\right)$—Represents the fail test cases associated with the statement, where stat has covered;

${pass\prime }_{tc}\left(stat\right)$—Represents the passed test cases associated with the statement, where stat has covered;

The rank is determined through the harmonic mean of two factors, i.e., increase identified by α(stat), and sensitivity identified by β(stat), where α(stat) and β(stat) are the levels of predicate stat and differentiate the feature distributions of predicate stat for unsuccessful executions across all implementations that are cover over stat.

#### 3.2.3. Program Snippet Analysis

The program snippet analysis is performed over the behavioral aspect of the program on executing a test case for analyzing the response in the run-time and stack trace. If the target parameter is inaccurate, it may affect some other parameters in the software. In the same way, statements with parameters or the literals in the same snippet have the potential to be fault-inducing expressions as well. Each statement’s vulnerability rank is directly proportional to how often it normalizes incorrect statements. Snippet analysis may be used if there is just one failed test case. The statement that fails is used as the starting point, and then the rest of the statements are stripped off from it.

Throughout implementation, if a statement is erroneous, the program will raise an exception. The collection of active stack sessions throughout a problematic program’s execution is also one of the most helpful debugging strategies [27,28]. The code snippet and associated test cases used in the validation are being presented in the Table 3 for better comprehensibility of the current study. 

The tick mark represents the pass of the test case associated with the corresponding line of the code snippet, and the cross symbol represents the test case’s failure. Based on the failed test cases, the corresponding statement is given the vulnerability ranking.

### 3.3. Feature Set Scaling and Initial Ranking

A feature scaling approach is used to standardize the overall range of features set in input data. The feature set in the input program includes varying values throughout the learning phase while minimizing the loss function. Scaling is performed over iterations to make the localization algorithm reach the global or local best fast and precisely. In the current study, the min-max normalization is performed in scaling the feature values in the range 0–1 [29]. The normalizing approach known as Min-Max brings numerous advantages over traditional scaling techniques. Min-Max scaling is capable of handling the non-Gaussian feature distribution. Min-Max normalization is made to solve the loss of precision in a method that optimizes the gradient while moving toward the global solution. It produces target values ranging from 0 to 1 by taking the column’s min and max values, as illustrated in Equation (5):

(5) $$f_{new}=\frac{f-f_{min}}{f_{max}-f_{min}}$$

From Equation (5), the variable $f_{new}$ denotes the new normalized values in the range 0–1, $f_{min}$ denotes the smallest values associated with the corresponding feature and the variable $f_{max}$ denotes the largest value associated with the particular feature. The variable f denotes the corresponding data sample. Now for fine-tuning the ranks of each of the features that are identified, the ranking is performed in concern to the global best, resultantly the vulnerability rank of the current feature is following the other features across the complete program, and it does the job of localizing the bugs based on the impact of the bug on the software [30,31].

(6) $$stat_{Rank}=stat_{Rank-1}+G\_Best\_stat_{Rank}-L\_Best\_stat_{Rank-1})$$

From Equation (6), the variable$\;stat_{Rank}$ denotes the vulnerability rank of the statement at the corresponding iteration and the variable and$\;stat_{Rank-1}$ denote the rank associated with the corresponding statement in the previous iteration. The variable $G\_Best\_stat_{Rank}$ denotes the global best vulnerability ranking across the complete program and similarly the variable $L\_Best\_stat_{Rank-1}$ denotes the local best vulnerability rank within the code snippet associated with the software program. The ranks on updating concerning the global best rank, the more vulnerable statements through the program are prioritized to resolve first before the less significant statements.

## 4. Proposed Model

**Figure 2:**
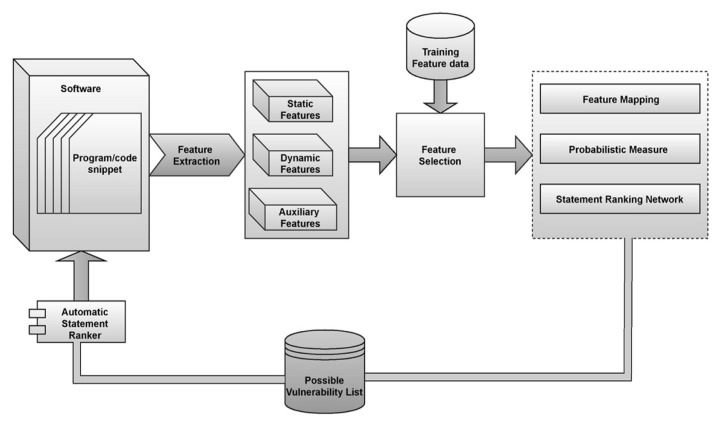
Represents the block diagram of the proposed fault localization model.

The proposed ABNR model works are mechanized to identify the features based on the significance using the self-attention layer through the weighted approach, and aggregation is performed in the neural ranking process. The statements are then ranked based on the severity of the vulnerability using the softmax layer of the neural ranking model [32]. The block diagram of the proposed model is presented in Figure 2 [33]. The code snippets that are the part of the software program are fed as the input for the neural ranking model. The neural ranking model identifies the features from the data and assigns the weights to the features based on the importance of those feature in the fault determination. The features are then correlated with the features that are already trained to the model using the feature map and the probabilities are assessed. Then based on the probabilities the ranks are provided to the statements.

### 4.1. Layered Architecture of the Aggregation-Based Neural Ranking

The layered model of the ABNR neural ranking model consists of multiple layers that would determine the rank of the statement associated with the vulnerability. The model architecture includes the self-attention layer, neural ranking layer through aggregation, and the SoftMax layer approximating the suitable rank for the statement. The model is trained with the data with similar statements with relevant vulnerabilities.

Neural Ranking techniques have subsequently been presented for determining the relevance of a susceptible statement to a code snippet by examining the vulnerability statements, patterns of statement fragments matching in the training data, or a combination of the two. By seeing a huge variety of vulnerable and normal code samples during training, these models often learn to discriminate between the code feature distributions associated with a pertinent and a less pertinent vulnerable statement-code snippet combination. Compilations of statements in each category provide a partial order for the statements in that list. Assigning a numeric score for every such statement usually induces the kind of vulnerability ranking. The neural ranking technique aims to rank program statements in an unseen lists manner comparable to how scores were determined from the training samples.

#### Self-Attention Layer

The self-attention layer is mechanized to integrate several feature sources and allot suitable weights to each source of information for fault localization. The neural ranking model assigns the erroneous statement the maximum rank. Attributed to the reason that various features have varying degrees of significance, the self-attention layer is utilized to integrate and improve important data from static and dynamic features. The normalization layer separates all three kinds of features, semantic, statistical, and dynamic. The self-attention layer combines all three vectors and assigns the vector to the f(x),g(x), and h(x) components [33]. Each layer would subsequently distinguish inputted features and produce a feature map. Self-attention connects data from various places within input pattern to compute the scaled dot product attention, as shown in Equation (7):

(7) $$attention\left(f\left(x\right),g\left(x\right),h\left(x\right)\right)=softmax\left[\frac{{\left(f\left(x\right)\times g\left(x\right)\right)}^{T}}{\surd d_{g\left(x\right)}}\right]\times h\left(x\right)$$

(8) $$f\left(x\right)={\mathbb{R}}^{f_{i}\times f_{d}}$$

(9) $$g\left(x\right)={\mathbb{R}}^{g_{i}\times g_{d}}$$

(10) $$h\left(x\right)={\mathbb{R}}^{h_{i}\times h_{d}}$$

**Figure 3:**
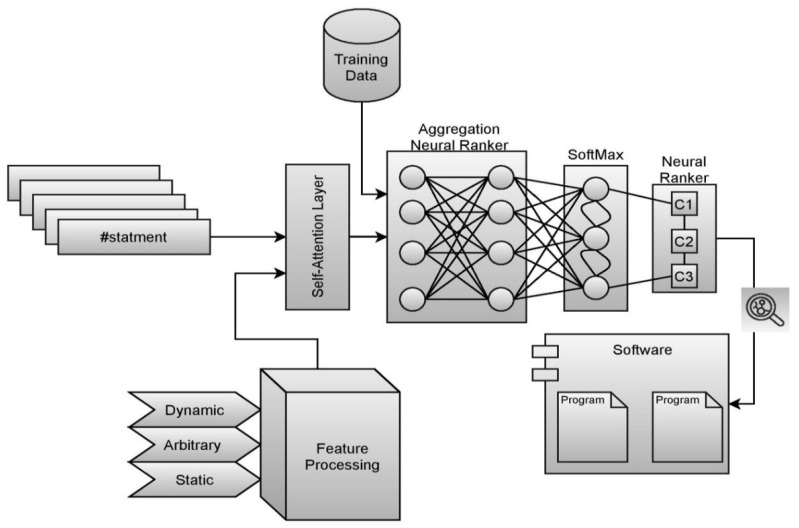
Represents the layered architecture of the fault localization model.

From the above Equations (7)–(10), the components f(x), g(x), h(x) are the components associated with statements, test cases, and values, respectively. The variables $f_{i},g_{i},h_{i}$ denote the ith element of each of that feature, and the variables $f_{d},g_{d},h_{d}$ denote the dimension associated with each of those features. The variable d denotes the spatial dimension with finite set of features. The value $\surd d_{g\left(x\right)}$ divides the dot product of the snippets with the possible vulnerable statements, and softmax is applied to obtain the associated weights of each such feature. Earlier studies hypothesize that when d increases, the amplitude of the dot product increases, forcing the softmax into areas with very tiny gradients. To compensate for this impact, multiply the dot products by $\frac{1}{\surd d_{g\left(x\right)}}$ that avoid softmax converging toward less significant features. Typically, every statement’s rank is calculated as a weighted sum of associated values, for each weight of the value being determined by an objective statement for the statement using a homologous test case [34]. The layered architecture of the proposed model is presented in Figure 3.

### 4.2. Weights Assessment Procedure

Weighing characteristics to enhance class labeling accuracy is possible when various factors affect the class label differently. The normalized mutual information (NMI) [35] between each feature and the class label as the feature’s weight since mutual information measures the independence of two variables. To better understand the NMI-based weight assignment over the dataset D with T instances, which comprises two parameters p and q with m,n instances, respectively, over the class label c [36]. The weights for both the features are determined through the following Equations (11) and (12):

(11) $$\omega \left(p\right)=\frac{\alpha \left(p,c\right)}{mean\left(\beta \left(p\right),\beta \left(c\right)\right)}$$

(12) $$\omega \left(q\right)=\frac{\alpha \left(q,c\right)}{mean\left(\beta \left(q\right),\beta \left(c\right)\right)}$$

From Equations (11) and (12), the variable α denotes the mutual information. Furthermore, the variable β denotes the entropy. The mutual information is determined through the Equations (13) and (14):

(13) $$\alpha \left(p,c\right)=\overset{m-1}{\underset{i=0}{\sum }}\overset{k-1}{\underset{j=0}{\sum }}\frac{\left|p_{i}\cap^{\;}\;c_{j}\right|}{T}\log\;\left(\frac{T\left|p_{i}\cap^{\;}\;c_{j}\right|}{\left|p_{i}\right|\left|c_{j}\right|}\right)$$

(14) $$\alpha \left(q,c\right)=\overset{m-1}{\underset{i=0}{\sum }}\overset{k-1}{\underset{j=0}{\sum }}\frac{\left|q_{i}\cap^{\;}\;c_{j}\right|}{T}\log\;\left(\frac{T\left|q_{i}\cap^{\;}\;c_{j}\right|}{\left|q_{i}\right|\left|c_{j}\right|}\right)$$

Entropy, as it pertains to machine learning, is a measure of the unpredictability and randomness of the analyzed information. The bigger the entropy, the more difficult it is to make any inferences out from the information. The entropy is being assessed through the Equations (15)–(17).

(15) $$\beta \left(p\right)=-\overset{m-1}{\underset{i=0}{\sum }}\rho \left(\frac{\left|p_{i}\right|}{T}\right)\log\left(\frac{\left|p_{i}\right|}{T}\right)$$

(16) $$\beta \left(p\right)=-\overset{m-1}{\underset{i=0}{\sum }}\rho \left(\frac{\left|p_{i}\right|}{T}\right)\log\left(\frac{\left|p_{i}\right|}{T}\right)$$

(17) $$\beta \left(c\right)=-\overset{m-1}{\underset{i=0}{\sum }}\rho \left(\frac{\left|c_{i}\right|}{T}\right)\log\left(\frac{\left|c_{i}\right|}{T}\right)$$

### 4.3. Aggregation Based Neural

The aggregation-based neural ranking model for the bug localization is robust in assigning the ranks to the statements by considering the other statements within the code snippet. The principle logic in the model is that if any of the statements are erroneous, the consecutive statements after that are influenced by the erroneous statement. Resultantly the scores of the statements are updated following the previous nodes along with their vulnerability score. However, the scores of the statements are updated on normalizing each statement and correcting them where so ever required. The mathematical model for the same is presented in the current subsection of the study.

To better understand the process flow of the fault localization model, consider the following diagram, which has four statements in the snippet labeled *A*, *B*, *C*, and *D*, in which the erroneous origin statement is depicted by *A*. Statement *D* represents the data-receiving destination. It is believed that the intermediate statements are located between the statements and the nodes intended to be reached by the data. The fault rank is used to determine the vulnerability associated with each corresponding statement. This procedure is described in detail in Equations (18)–(23).

(18) $$R\left(S_{A}\right)=\left\{\left(Int_{\omega }\left(A\right)\times \rho \left(A\right)\right)+\Delta \left(\omega_{A,B}+\omega_{A,C}+\omega_{A,D}\right)\right\}$$

(19) $$R\left(S_{B}\right)=\left\{\rho \left(B\right)-\Delta \left(\omega_{A,B}+\omega_{B,C}+\omega_{B,D}\right)\right\}$$

(20) $$R\left(S_{C}\right)=\left\{\rho \left(C\right)-\Delta \left(\;\omega_{A,C}-\omega_{B,C}+\omega_{C,D}\right)\right\}$$

(21) $$R\left(S_{D}\right)=\left\{\rho \left(D\right)-\Delta \left(\omega_{A,D}-\omega_{B,D}-\omega_{C,D}\right)\right\}$$

From Equations (18)–(21), various variables are being used to demonstrate the ranking procedure by aggregating the weights associated with each statement.

$R\left(S_{A}\right)$—Rank associated with statement *A*;

$R\left(S_{B}\right)$—Rank associated with statement *B*;

$R\left(S_{C}\right)$—Rank associated with statement *C*;

$R\left(S_{D}\right)$—Rank associated with statement *D*;

$Int_{\omega }\left(A\right)$—Initial weights associated with node *A*;

ρ(A)—Vulnerability probability of statement *A*;

ρ(B)—Vulnerability probability of statement *B*;

ρ(C)—Vulnerability probability of statement *C*;

ρ(D)—Vulnerability probability of statement *D*;

Δ—The overall difference among the weights associated with statements;

$\omega_{i,j}$—Weigh the difference among the statements ‘*i*’ and ‘*j*’.

**Figure 4:**
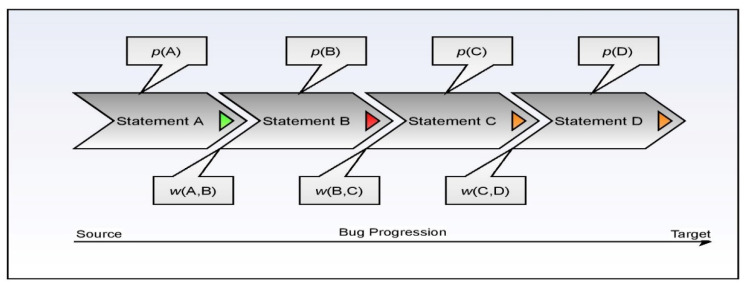
Represents the aggregative score model for fault localization.

The aggregation-based ranking approach is reasonably fair in approximating the vulnerability score is concerning the other statements within the same code snippet. The initial probability is approximated through the softmax scores associated with the statement. The working procedure of the aggregation model is demonstrated in Figure 4.

The sample test case for evaluating the division of two numbers is presented through the Algorithm 1. The outcome of the test case would be a pass or fail based on the provided data. The test case would be evaluated against the data, and many such test cases are associated with each such code snippet for evaluating the vulnerability score of the statement.

**Algorithm 1** (Algorithm of sample test cases)

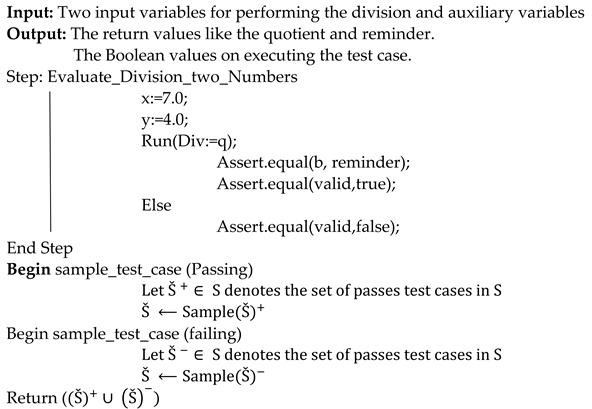



### 4.4. Dataset Acquisition and Experimental Setup

Siemens test case topic applications [37] and four Unix utility programmed like gzip, sed, flex, and grep are considered to assess the proposed fault localization model [38]. The data are the part of Typically, and many researchers have used these theme programs for fault localization. Siemens test case programs are used for one fault, whereas Unix utility applications include actual and injected flaws obtained from [39]. Each topic program in the Siemens validation set contains around 1000 test inputs and comprises seven distinct test programs: tcas, tot info, schedule2, print tokens, print tokens2, replace, and tot info2. File compression and decompression are handled by the gzip program in the Unix real-life utility program. Name files may benefit from the program’s ability to shrink their size, which is why it is often used. The gzip program’s input consists of 13 different parameters and a list of files to compress. With 6573 lines of code and 211 test inputs, the software can do a lot. The sed software is used to make small modifications to a stream of input. It is mostly used for parsing text input and making user-specified modifications to that data. There are 360 test inputs and 12,062 lines of code in the program.

A lexical analyzer is what the flex program does. The input files were created of rules, which are collections of regular expressions and C code. There are 13,892 lines of code in all, and 525 test inputs are provided. Patterns and files are the two input parameters for the grep program. Every file that includes a match to one of the patterns has lines printed by the software. Lines of code: There are 12,653, and 470 inputs provided [40]. Real and injected errors will now be included in Unix utility applications. The details of the subject programs considered in the proposed model evaluation are presented in Table 4 [41,42]. The normal and vulnerable program statements are the samples that are used in the both the training and the validation phase of the proposed model. Training data contains 60% of the original data samples of the code. The overall samples are indeed made up of a 60% training dataset and a 40% validation dataset chosen at random from normal and defective samples. The remaining 40% of the original dataset is the testing dataset assumed as the unseen portions used to assess the ABNR fault localization performance. 

The performance of the proposed ABNR model is evaluated by executing multiple test cases over the code snippets. The test cases assess the validity of the code snippet under variable factors including the divergent inputs and operational conditions. The evaluations are carried locally in the standalone computer by deploying the necessary software. The details of the experimental environment where the experimentation is carried is presented in Table 5.

## 5. Results and Discussion

To assess a defect localization approach’s efficiency, it is critical to use appropriate measurements. The aggregate number of assertions is evaluated, Wilcoxon signed-rank test and Top-N are all used in the current study. If it is to be determined, an appropriate measure should be employed to assess the relevance and usefulness of a defect localization method. The score is a criterion that is defined as the proportion of code snippet that does not need to be inspected to identify a flaw in the program. The exam score is defined as the percentage of code that has to be inspected until the first statement in which the problem is located is reached during the examination process. The exam score (ES) is often used in many research to assess the efficacy of a fault localization method [43,44]. A method α with a lower exam score than that of another approach β will be regarded as being more successful in comparison to technique B since technique α requires fewer code statements to be inspected to identify any flaws than technique β. The *ES* values are mathematically evaluated using the Equation (22), concerning the vulnerability score of the statement recognized by $V_{s}\_Stat$.

(22) $$ES=\frac{V_{s}\_Stat}{Num\_of\_lines\_of\_code}$$

The cumulative number of statements that must be evaluated concerning subject programs to identify errors is taken into account [45]. So, for any given programs with n faulty versions, *A(k)* and *B(k)* are the percentages of statements that must be reviewed for two fault localization techniques, *A* and *B*, to detect all defects inside the kth Faulty version of the program. Approach *B* is more effective than technique *A* when it requires a programmer to analyze fewer statements to find all flaws in the erroneous versions, as shown in Equation (23); procedure B seems to be efficient over procedure A.

(23) $$\overset{n}{\underset{k=1}{\sum }}B\left(k\right)<\overset{n}{\underset{k=1}{\sum }}A\left(k\right)$$

In addition, the Wilcoxon signed-rank test [46] is used to provide a rigorous empirical assessment of methodologies in terms of their efficacy. Top-N represents the proportion of errors within Top N (N = 1, 5, 10) places that a fault localization method ranks for any problematic code snippet. As a result, the measure becomes stronger as the magnitude of N in Top-N decreases [47]. The performance of the proposed model is compared against the similar fault localization models Jaccard [48], Ochiai [48,49], Tarantula [48,50], software-network centrality measure (SNCM) [42], fault localization technique based on complex network theory (FLCN-S) [40]. The total amount of statements evaluated in Siemens’ validation set and Unix utility applications to find errors are presented in Table 6.

**Figure 5:**
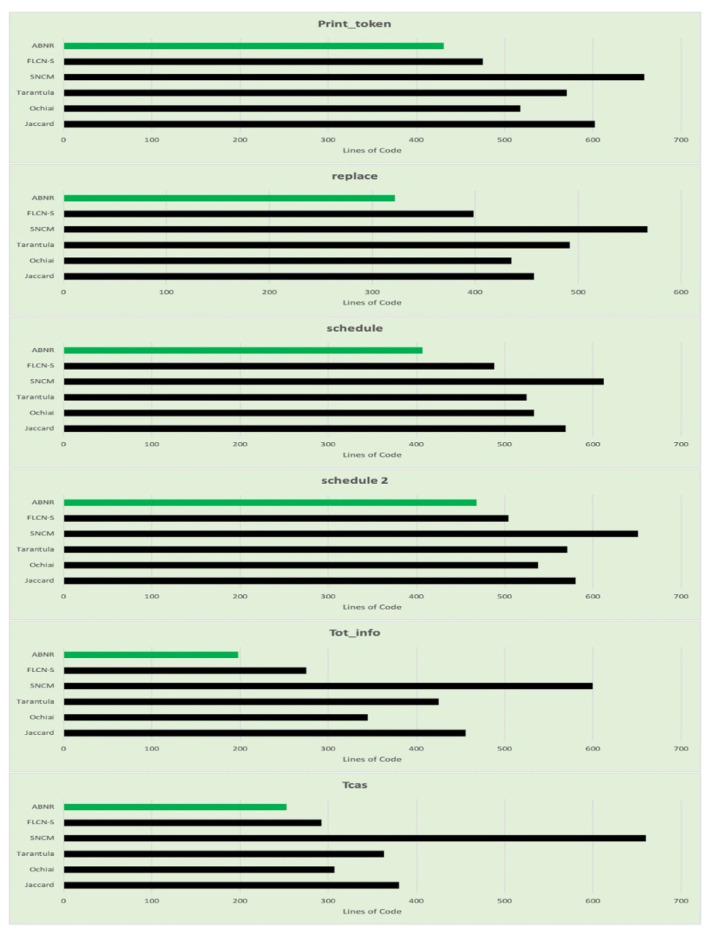
Graphs representing the number lines of code evaluated for fault localization for each category in Siemen’s validation set.

The proposed ABNR model outperforms compared to the other existing models for fault localization. The performance is determined based on the number of statements being evaluated to determine the faulty code snippet. The model capable of recognizing the vulnerability with fewer statements is assumed to be efficient with lesser computational efforts and faster response. From Table 6, it can be observed that the proposed model is comparatively better than the other model considered. The graphs are generated from tabular data in Figure 5.

**Figure 6:**
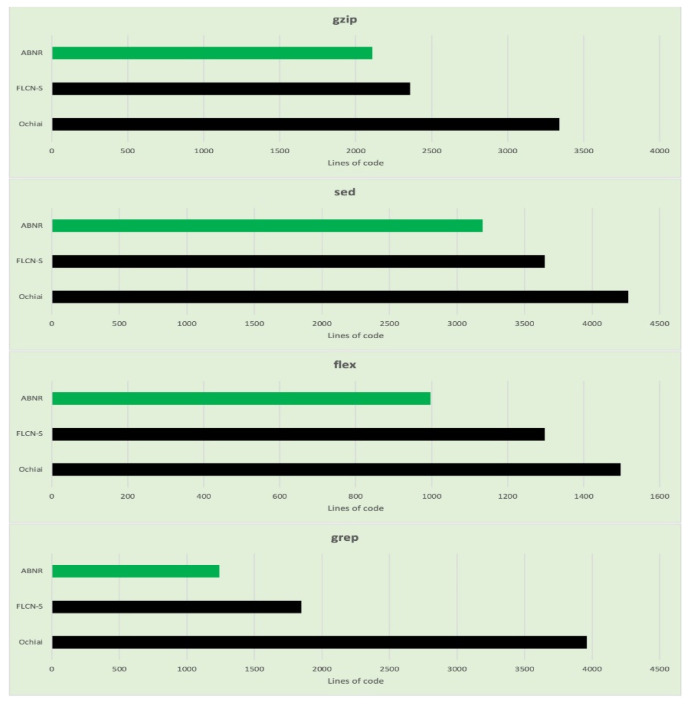
Graphs representing the number lines of code evaluated for fault localization for each category in the Unix utility program.

The performance of the proposed ABNR model is also evaluated against the Unix utility application programs. The model has recognized the faulty statements in the code snippet by evaluating the fewer lines of code, which needs comparatively lesser computational efforts and is much faster than its counterparts. The details about the number of lines of code evaluated for Unix applications are presented in Table 7, and the corresponding graphs are presented in Figure 6.

**Figure 7:**
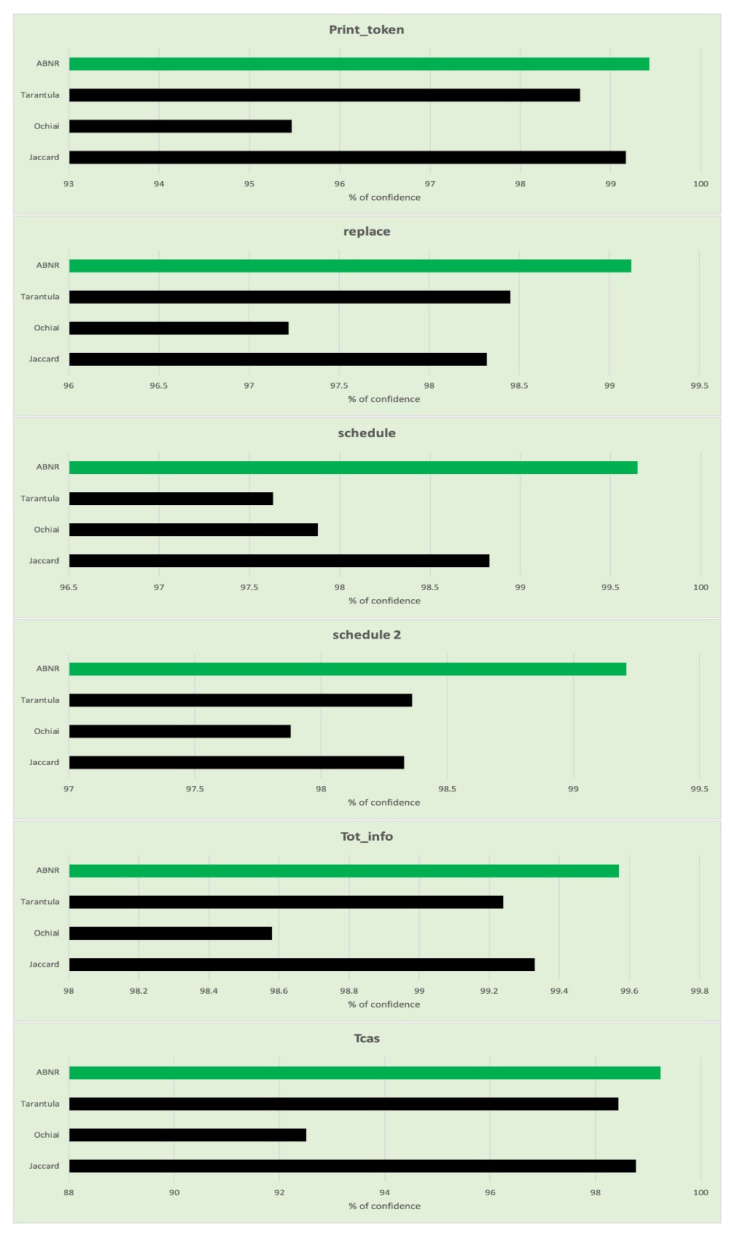
Graphs representing the confidence level of fault localized for each category in Siemen’s validation set.

It can be observed from the values presented in Table 6 and Table 7, that the proposed aggregation-based neural ranking model performs as desired by assigning the ideal ranks to the vulnerable statements that would assist in fault localization by verifying the fewer statements. The proposed model is further evaluated through the Wilcoxon signed-rank test concerning the confidence value of similarity among two independent samples of code to compare overall sample average rankings of two samples using paired differential tests. The ABNR has been evaluated like the other models such as Jaccard, Ochiai, Tarantula. The comparative analysis of the confidence measures is presented in Table 8, and Figure 7 represents the graphs generated from the evaluated confidence values. The proposed ABNR model has exhibited better confidence in evaluating the fault statements in the code snippet.

**Figure 8:**
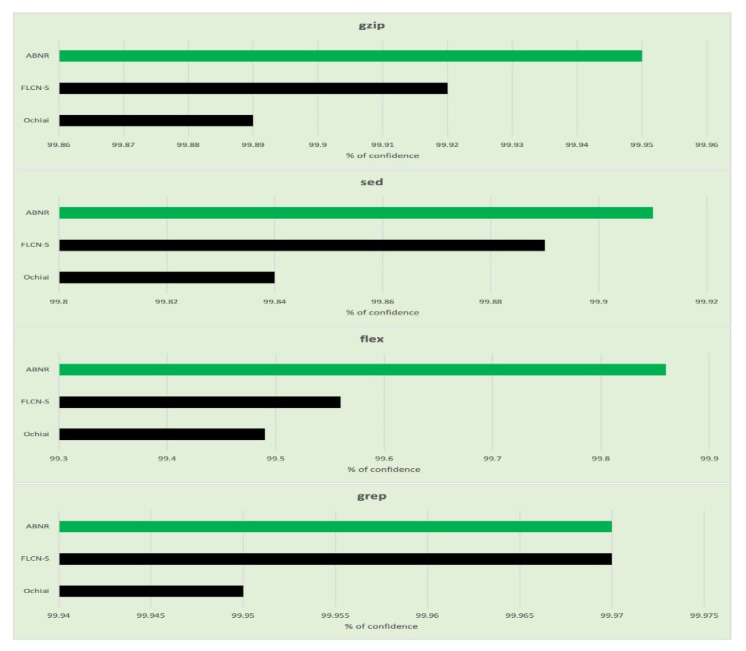
Graphs representing the confidence level of fault localized for each category in Unix utility applications.

The Wilcoxon signed-rank test is performed over the Unix application programs to assess the confidence of similarity among two independent code samples. The assessed values for the same are presented in Table 9, and the generated graphs are presented in Figure 8.

The efficiency of the Fault localization approaches is evaluated based on their ability to locate actual flaws. They also perform significantly better at incorporating the right response over top N statements of their outcome. Usually, N would be 5 or 10. The study has found that concentrating on factors other than location would lead to substantial advancements in fault localization. The assessed values concerning Top-N statements are presented in Table 10.

It can be observed from the above table that the performance of the ABNR is better compared to that of the counterparts as the proposed model is capable of localizing the vulnerable statements more appropriately compared to the other approaches. The evaluations on the proposed model have made it clear that the model is more precise with lesser computational efforts in fault localization.

### 5.1. Practical Implication

**Figure 9:**
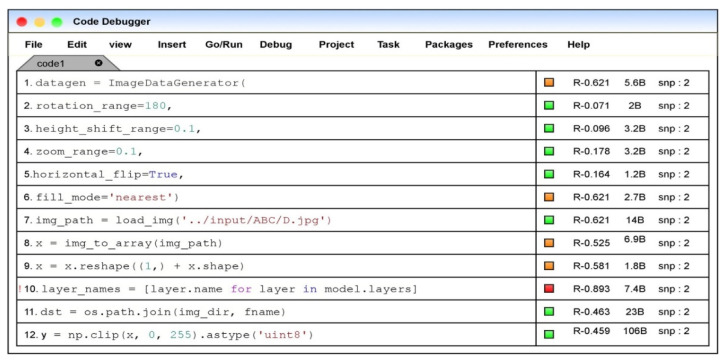
Image of the code editor with fault localization model.

The proposed fault localization model through the aggregation-based neural ranking method is incorporated in the code editor names code debugger to evaluate the faults in the code snippet. The fault localization model would update the ranks for each corresponding statement in the code snippet on evaluating the code. The color box represents the severity of the error or bug corresponding to the code. The orange color denotes the possible fault or the vulnerability associated with the code statement, and the red color represents the faulty statement. The green color represents the error-free statements, and the variable “snp” denotes the snippet of the corresponding program. Figure 9 denotes the front end of the practical implication model.

**Figure 10:**
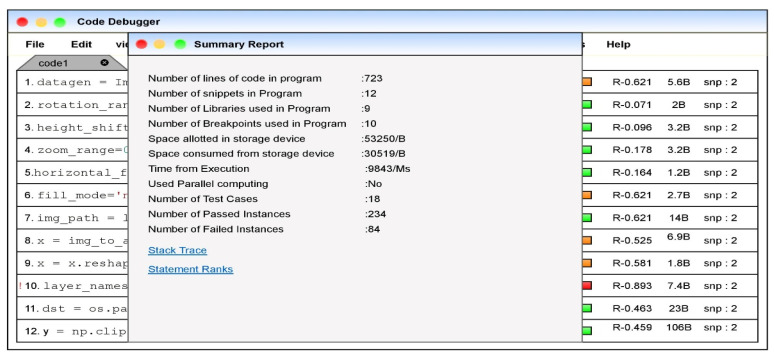
Image summarized report about the code snippet.

The dashboard embedded in the dashboard would be immediate assistance for the developer to assess the statement’s vulnerability. The summary module is associated with the editor that would conclude the metadata associated with the code snippet executed. The developer can obtain the summarized information about the code snippet at a single point. Figure 10 that is presented below denotes the summarized information about the code snippet.

The proposed technology for fault localization integrated into the editor would assist the developer in faster fault localization. Upon successfully coding a function in the program or the code snippet of an application, the generated summarized report would assist the developer in ease of debugging the program.

### 5.2. Threats to Validity

The proposed ABNR model is confined to a single error approximation and assigns the ranks to the statements; all the statements that come after the vulnerable statements are influenced by the erroneous statements. But the ABNR is limited for assessing the ranks from the single statement error that can be further improvised by multi-error prediction mechanism. The multi-error prediction model would assist in precise rank of the statements for better localization probability.

## 6. Conclusions

The current study proposes a novel and efficient bug localization approach through an aggregation-based neural ranking technique. The proposed model considers the vulnerable statement, and ranking is provided to all the consecutive statements based on the faulty statement, which will assist the developers in quickly and precisely pointing out the vulnerability. The proposed approach on evaluating the benchmark subject programs like the Siemens validation set and Unix Utility applications shows reasonable performance than the counterparts. The model is evaluated across the divergent metric like the assertions evaluated, Wilcoxon signed-rank test, and Top-N. The results have proven that the ABNR model can localize the faults by evaluating fewer statements with better confidence. The weights optimization would yield better performance, and incorporating the auxiliary memory components for maintaining the state information would enhance the performance of the fault localization model. The practical implication model shown in the results and discussion section is expected to be the common feature for the majority of the code editors in the future generation. Yet, there is good scope for research in assessing the overall rank of the code snippet based on the faulty statements, which would determine the overall quality of the software.

## Figures and Tables

**Table 1:** Table representing the categories of various parameters.

Parameter	Static Parameter	Dynamic Parameter
Access(method, attribute)		✓
Call(method, method)		✓
Consist(class, attribute)	✓	
Consist(class, method)	✓	
Consist(class, sub-class)	✓	
Consist(package, class)	✓	
Instance(object, class)		✓
Knows(object, object)		✓
Refers (method, method)	✓	
Refers(method, Attribute)	✓	
Sub-class(class, class)	✓	
Stack Trace		✓
Dynamic Program Splitting		✓

**Table 2:** Table representing the static features associated with the software.

Feature	Description of the Feature
#n_Lines	Total number of lines of code in the complete software program
#s_Lines	Total number of lines of code corresponding to snippet
#indt	Determines the level of indentation
#n_comments	Number of comments in the complete program
#s_comments	Number of comments in the corresponding code snippet
#n_anno	Number of annotations in the complete program
#s_anno	Number of annotations in the corresponding code snippet
#n_label	Number of labels in the complete program
#s_label	Number of labels in the corresponding code snippet
#n_g_var	Number of Global variables in the complete program
#n_l_var	Number of Local variables in the complete program
#s_g_var	Number of Global variables in the corresponding code snippet
#s_l_var	Number of Local variables in the corresponding snippet
#n_opr	Number of operators in the complete program
#s_opr	Number of operators in the corresponding code snippet
#n_kyw	Number of keywords in the complete program
#s_kyw	Number of keywords in the corresponding code snippet
#n_null	Number of null values in the complete program
#s_null	Number of null values in the corresponding code snippet
#n_tok	Number of tokens in the complete program
#s_tok	Number of tokens in the corresponding code snippet

**Table 3:** Table representing the code snippet with the outcome of test cases.

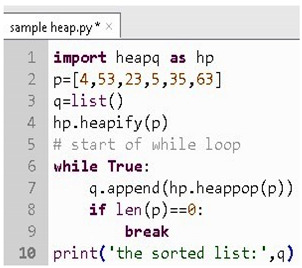	**TC-1**	**TC-2**	**TC-3**	**TC-4**	**TC-5**
	✓	✓	✓	✓	✓
	✓	✘	✓	✓	✓
	✓	✓	✓	✓	✓
	✓	✓	✘	✘	✓
	!	!	!	!	!
	✓	✓	✓	✓	✓
	✓	✓	✓	✓	✘
	✓	✓	✓	✘	✘
	✓	✓	✓	✓	✓
	✓	✓	✓	✓	✓

**Table 4:** Table representing the subject programs for evaluation.

Program	Number of Vulnerable Snippets	Number of Lines in the Code	Role	Test Cases
print_token	7	565	Lexical analyzer	4130
replace	32	412	Sequence replacement	2650
schedule	10	307	Priority scheduler	2710
tcas	41	173	Altitude separation	1608
Sed	7	12,062	Text manipulator	360
tot_info	23	406	Information Measure	1052
Gzip	5	6573	Data compression	211
Flex	22	13,892	Lexical analyzer	525
Grep	7	12,653	Sequence searcher	470

**Table 5:** Information about the implementation environment.

Environment Details	Specification
Operating System	Microsoft Windows 10
Processor	Intel Xeon E5-2687W
Architecture	64-bit
Memory allotted	720MB
GPU	NVIDIA Quadro P1000
Cuda-Parallel Processing cores	640
Language	Python
Libraries used	Pandas, Numpy, Scikit

**Table 6:** Denote the number of statements analyzed for fault detection from Siemen’s validation set.

	print_token	replace	schedule	schedule 2	Tot_info	Tcas
Jaccard	602	457	569	580	456	380
Ochiai	518	435	533	538	345	307
Tarantula	570	492	525	571	425	363
SNCM	658	567	612	651	600	660
FLCN-S	475	398	488	504	275	292
ABNR	431	322	407	468	198	253

**Table 7:** Denotes the number of statements analyzed for fault detection in Unix utility applications.

	gzip	sed	flex	grep
Ochiai	3342	4269	1497	3959
FLCN-S	2357	3651	1298	1848
ABNR	2109	3189	997	1241

**Table 8:** Denote the confidence level of fault detection from Siemen’s validation set.

	print_token	replace	schedule	schedule 2	Tot_info	Tcas
Jaccard	99.17	98.32	98.83	98.33	99.33	98.77
Ochiai	95.47	97.22	97.88	97.88	98.58	92.5
Tarantula	98.66	98.45	97.63	98.36	99.24	98.43
ABNR	99.43	99.12	99.65	99.21	99.57	99.23

**Table 9:** Denote the confidence level of fault detection from Unix utility applications.

	gzip	sed	flex	Grep
Ochiai	99.89	99.84	99.49	99.95
FLCN-S	99.92	99.89	99.56	99.97
ABNR	99.95	99.91	99.86	99.97

**Table 10:** Denote the Top-N assessment for faults localized in Unix utility applications.

	Top-N	gzip	sed	flex
Ochiai	5	29.15	32.82	29.11
	10	44.27	40.67	39.12
FLCN-S	5	35.62	41.01	48.01
	10	51.01	49.00	58.44
ABNR	5	41.21	48.27	54.11
	10	57.89	54.20	66.02

## Data Availability

The data for evaluation of the model is being acquired from an open-source repository, namely Software-artifact Infrastructure Repository (SIR), that consist of the subject programs like print token, schedule, schedule 2, replace, tcas, tot_info that are part of Siemen’s validation set and programs like gzip, sed, flex, and grep that are the part of the Unix utility suite. The programs are associated with the test cases that would assist in tracing the vulnerable statements.
